# Can voluntary attention control be elicited and detected in the lab? Preliminary evidence for a dual-path model linking intention to agency

**DOI:** 10.3758/s13414-025-03087-6

**Published:** 2025-05-20

**Authors:** Bradley S. Gibson, Jamie M. Trost, Lijuan Wang

**Affiliations:** Department of Psychology, University of Notre Dame, Notre Dame, IN 46556 USA

**Keywords:** Voluntary attention control, Agency, Visual search, Ironic mental processes

## Abstract

Individuals orient their attention in a voluntary fashion when they willfully shift their attention in the visual field. Volitional actions can only be elicited in the lab, however, when experimental paradigms allow sufficient expression of agential capacity. Unfortunately, the standard paradigm for eliciting voluntary attention control (VAC) does not appear to allow such expression. The present study therefore attempted to increase the elicitation of VAC by using a modified spatial cueing paradigm that granted participants greater freedom in choosing the direction of the cue. It also attempted to improve the detection of VAC by using statistical mediation analyses to examine the relations between measures of intention, agency, and performance, as well as how the magnitude of these relations might be moderated by three cue validity contexts (100%, 70%, and 25%). Based on a total sample of 720 participants, the present findings showed that the “total effect” of intention on agency can be decomposed into two paths. The “direct effect” of intention on agency generally reflects VAC in that increases in intention were associated with increases in agency, but only in the 100%-valid cue context. However, the “indirect effect” of intention on agency passes through performance, and it reflects a process that appears to be more experience based and less volitional. Altogether, the present study recommends new methods for eliciting and detecting VAC in the lab while also exposing some shortcomings in more traditional measures of VAC based on performance.

Individuals are thought to act in a voluntary fashion when they are able to express three agential capacities: (1) the capacity to generate behavioral options to consider; (2) the capacity to decide on one of those options; and (3) the capacity to causally control the execution of the chosen behavior (List, 2019; see also Sheldon, 2024). Unfortunately, allowing research participants to manifest any of these three agential capacities is typically unwelcomed in the psychology lab because experimental psychologists often want to control both possible and enacted behaviors within the experimental context as much as possible. As Sir Ronald Fisher (1935/1971) wrote, “Experiments are just experiences that are carefully planned [by experimenters] in advance” (p. 8). Because Fisher’s notion of the scientific method was explicitly designed to limit agential capacity, some have questioned whether a lab-based, empirical science of volitional action is even possible (see, e.g., Howard & Conway, 1986).

In the present study, we focused exclusively on a specific type of volitional action known as voluntary attention control (VAC), and we examined the extent to which VAC can be elicited and detected in the lab. Individuals orient their visual attention in a voluntary fashion when they willfully shift their attention in the visual field, usually to accomplish some goal (Nadra & Mangun, 2023). At first glance, this effort may seem unnecessary to many attention control researchers because, contrary to the concern raised above, VAC has been largely taken as established fact in the attention control literature (see, e.g., Theeuwes, 2018; for further discussion, see Gaspelin & Luck, 2018; Wolfe, 2018). However, we contend that the standard methods used to *elicit* VAC in the lab are inadequate because they were not explicitly designed to elicit any of the three agential capacities listed above (Gibson et al., 2023a, 2023b). Moreover, we contend that the standard behavioral measures used to *detect* VAC are based mainly on assumptions that have not been adequately tested. Consequently, we contend that much of what we think we know about VAC may be incorrect. Thus, in our opinion, the extent to which VAC can be elicited and detected in the lab must be reexamined. In so doing, we sought to pay tribute to Mary A. Peterson, who made seminal contributions to our understanding of the role of intention in perception and attention (see, e.g., Peterson & Gibson, 1991; Peterson & Hochberg, 1983).

## The standard approach to eliciting and detecting VAC in the lab

**Fig. 1:**
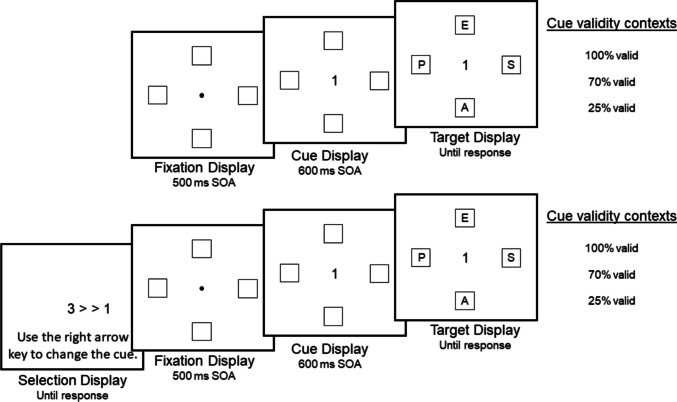
Example display sequences used in the spatial cueing paradigm. The top panel depicts the standard paradigm, and the bottom panel depicts the modified paradigm. In the modified paradigm, participants were given the option of changing the cued direction in the selection display. For instance, in the example above, the initial cue was “3,” and it was changed to “1,” which then appeared in the cue display. Note that participants had full control over the direction of the cue, but no control over the validity of the cue

The spatial cueing paradigm has been used extensively over the past several decades to elicit putative voluntary shifts of attention (Chica et al., 2014; Eriksen & Hoffman, 1972; Gibson & Kingstone, 2006; Gibson & Sztybel, 2014; Jonides, 1981; Posner, 1980; Posner et al., 1980). Consider, for example, the spatial cueing paradigm recently used by Gibson et al. (2023a) and shown in the top panel of Fig. 1. In this standard paradigm, participants performed a visual search task in which they were instructed to discriminate the identity of a target letter (*E* vs. *U*) that appeared among three distractor letters. In addition, they also instructed participants to use a spatial cue that appeared 600 ms before the target display to potentially help them find the target. Notice that the spatial cues used in this study were numbers ranging from 1 to 4, with the number 1 referring to the *top* location, the number 2 referring to the *right* location, the number 3 referring to the *bottom* location, and the number 4 referring to the *left* location. In some trial blocks, the spatial cue was 100% valid, which always helped participants find the target; in other blocks, the spatial cue was 70% valid, which mostly helped participants find the target; and in other blocks, the spatial cue was only 25% valid (chance), which mostly led participants astray.

Notice that, apart from consenting to voluntarily participate in the experiment, participants were not given any real choice about the direction in which they were asked to shift their attention in this paradigm; rather, the direction conveyed by the spatial cues was randomly determined by the experimenter and not by the participant. Nevertheless, shifts of attention that are elicited by the spatial cues in this paradigm have been assumed to be voluntary because participants can either choose to comply with task instructions and form an intention to orient their attention in accordance with the direction of the cue, or they can choose to forego task instructions, ignore the cue, and simply perform an unguided visual search for the only target present in the display (for further discussion, see Davis & Gibson, 2012; Pauszek & Gibson, 2016, 2018). Furthermore, there is a widespread assumption that standard manipulations of cue validity in the spatial cueing paradigm are sufficient to manipulate the strength with which participants form an intention to use the cue to find the target, with higher levels of spatial validity associated with stronger intentions to use the cue (Luck et al., 2021; Theeuwes, 2018). In this way, VAC is more likely to be elicited by highly valid cues than by uninformative cues.

Detection of VAC within the standard paradigm has been based mainly on a behavioral marker known as the “spatial cueing effect” (Posner et al., 1980), which was also evident in Gibson et al.’s (2023a) study: namely, participants performed the task faster when the target appeared at the cued (or valid) location than when it appeared at one of the noncued (or invalid) locations, suggesting that participants did use the cue to find the target. Moreover, the magnitude of this effect was larger for 70%-valid cues than it was for 25%-valid cues, suggesting that participants had a stronger intention to use the cues with higher spatial validity (note that this effect cannot be calculated in the 100%-valid cue context because there are no invalid trials). According to Theeuwes (2018), the spatial cueing effect that is elicited by informative spatial cues “is one of the most prominent examples of top-down attention that is truly volitional” (p. 4).

However, one complication here is that Gibson et al., (2023a, 2023b) found much weaker evidence for VAC within this standard paradigm when they attempted to directly measure participants’ ratings of their own agential capacity; individuals who act voluntarily tend to feel that their intentions are the primary cause of their actions (Bandura, 2001; List, 2019; Mitchell, 2023; Sapolsky, 2023; Wegner et al., 2017), and this feeling is reflected in the sense of agency (Firth, 2013; Synofzik et al., 2013; Tapal et al., 2017). That is, individuals tend to report a stronger sense of agency when they feel “in control” of their actions (Ashby et al., 2024; Metcalfe & Green, 2007).

Accordingly, Gibson et al., (2023a, 2023b) asked participants to self-report their sense of agency using a 7-point scale after they completed blocks of 40 trials in which they encountered a consistent level of cue validity. Given that participants’ intentions to find the target by using the cue were expected to be weaker as cue validity decreased, Gibson et al. hypothesized that the sense of agency should also decrease in a linear fashion as cue validity decreased. Surprisingly, the findings suggested that only approximately 60% of the participants generated the expected pattern of agency ratings. These unexpected findings prompted Gibson et al. (2023a) to consider whether the expression of agential capacity could be improved across participants by devising a modified spatial cueing paradigm.

## A new approach to eliciting VAC in the lab

Gibson et al. (2023a) attempted to increase the elicitation of agential capacity by allowing participants to freely choose one of the four cued directions on each trial (i.e., the second agential capacity listed above; for related attempts to increase agential capacity in other paradigms, see Bengson et al., 2014, 2015; Gmeindl et al., 2016; Hopfinger et al., 2010; Huffman & Brockmole, 2020; Nadra et al., 2023; Taylor et al., 2008; Wen & Haggard, 2018). As shown in the bottom panel of Fig. 1, this new paradigm was similar to the standard paradigm, except that it contained a selection display at the start of each trial. Similar to the standard paradigm, one of the four number cues appeared randomly in the selection display; however, unlike in the standard paradigm, participants in the modified paradigm were given the option of using the rightward arrow key on the keyboard to change the direction but not the validity of the cue. In other words, participants could scroll through the number cues one at a time until they found the cue they wanted (the scroll order was randomly determined on each trial). Once the cue was selected, the trial proceeded as usual in the standard paradigm. For instance, as shown in the bottom panel of Fig. 1, the initial cue was the number 3, but it was changed to the number 1 in the selection display, which dictated that the number 1 cue appeared later in the cue display. The main findings showed that the proportion of individuals who generated the expected pattern of agency ratings increased from approximately 60% in the standard paradigm to approximately 90% in the modified paradigm.

The findings obtained in Gibson et al.’s (2023a) modified spatial cueing paradigm are potentially important because they suggest that the participants’ agential capacity could be improved by giving them greater control over cued direction. However, despite these positive findings, it is still not clear the extent to which VAC was manifested in this paradigm. For instance, the pattern of spatial cueing effects, which has served as the primary behavioral marker for VAC in this paradigm, was very similar across the standard and modified paradigms, though it was found to be significantly larger overall in the modified paradigm.

Although the spatial cueing effect is a potentially reasonable basis for detecting VAC in the spatial cueing paradigm, the extent to which this effect (or any effect based on task performance) accounts for VAC has not been directly tested, in large part because there has not been an alternative measure of VAC with which to compare it. Moreover, the spatial cueing effect has typically been based on group averages within each cue validity context. Consequently, the extent to which the spatial cueing effect (or any effect based on task performance) serves as an adequate behavioral marker of VAC would benefit from more direct testing, and it would also benefit from testing these predictions at an individual difference level of analysis (Gibson et al., 2023a, 2023b). Indeed, one of the virtues of the modified spatial cueing paradigm is that it affords a potentially richer analysis of VAC because, in addition to providing performance-based measures, it also provides separate measures of intention and agency for each participant, which in turn can be used to construct a measure of VAC that is separate from performance.

## Statistical mediation as a tool for understanding VAC

**Fig. 2:**
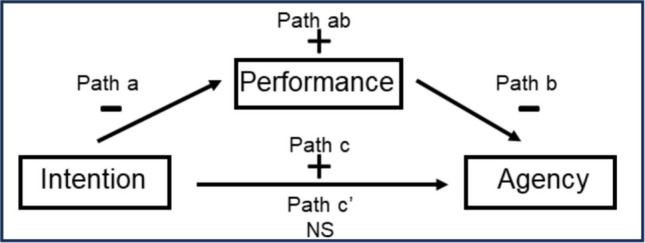
Example of the statistical mediation model used in the present study. The figure shows predicted relations between intention, performance, and agency ratings in the 100%-valid cue context. Path c represents the total effect of intention on agency. Paths a and b together represent the indirect effect of intention on agency that operates through performance, and path c′ represents the direct effect of intention on agency that remains after the indirect effect is controlled. Note: “plus” signs represent expected positive relations; “minus” signs represent expected negative relations; and “NS” represents expected zero relations

In the present study, we utilized statistical mediation models to advance understanding of VAC by examining specific relations between measures of intention, agency, and performance. The general model is depicted in Fig. 2; for the sake of brevity, we will consider only the 100%-valid cue context in our introduction of this model (though the 70%- and 25%-valid cue contexts will also be included in the results below). Generally speaking, the goal of statistical mediation analysis is to explain why two variables are related (MacKinnon, 2008). With respect to VAC, the most critical relation to explain is the one between intention and agency because individuals who act voluntarily tend to feel that their intentions are the primary cause of their actions (List, 2019). As is customary in statistical mediation analysis, we labeled this relation the “total effect” of intention on agency, which is also known generically as “path c” (see Fig. 2). In the 100%-valid cue context, we predicted that there should be a positive relation between intention and agency at the individual level (stronger intentions should be associated with stronger agency). Thus, we contend that this total effect of intention on agency is a basic manifestation of VAC in this paradigm. However, as we will discuss in greater detail below, we also predicted that the total effect of intention on agency should be moderated by the experimental manipulation of cue validity context because this manipulation reflects the amount of consistency between participants’ intentions and the external task context.

Assuming we can demonstrate a significant total effect of intention on agency, at least in the 100%-valid cue context, the next critical step is to examine the extent to which this effect might be explained by a type of third variable known as a *mediating* variable (MacKinnon, 2008). In the context of the modified spatial cueing paradigm, task performance (and specifically RT performance) is an obvious candidate for a mediating variable because (i) it intervened between the generation of intention at the start of each trial and the rating of agency at the end of each block (see Fig. 1), and (ii) it has served as the standard measure of VAC in the spatial cueing paradigm. As described in greater detail below, we will consider two different measures of RT performance (including the spatial cueing effect). For now, statistical mediation analysis examines the extent to which the total effect of intention on agency can be decomposed into two separate effects. On the one hand, the upper path shown in Fig. 2 reflects the “indirect effect” of intention on agency, which reflects the relation between intention and agency when it has been channeled through RT performance; on the other hand, the lower path reflects the “direct effect” of intention on agency, which reflects the adjusted relation between intention and agency after the effect of the indirect path has been controlled (or accounted for). The indirect effect can itself be broken down into two paths—referred to as “path a” and “path b”—and the direct effect is referred to as “path c-prime (c′).”

Examining the significance of the indirect effect is a critical first step in determining the extent to which the total effect can be explained by the mediator. In the present context, “path a” reflects the relation between intention and RT performance. In the 100%-valid cue context, we predicted that there should be a negative relation between intention and RT performance at the individual level (stronger intentions should be associated with smaller or faster RTs), though we also predicted that this relation should be moderated by cue validity context (see below for details). “Path b” reflects the relation between RT performance and agency. In the 100%-valid cue context, we also predicted a negative relation between RT performance and agency at the individual level (faster RTs should be associated with stronger agency), though once again we predicted that this relation should be moderated by cue validity context (see below for details). As is customary in statistical mediation analysis (MacKinnon, 2008), the overall significance of the indirect path is evaluated by multiplying the weights associated with “path a” and “path b,” and then determining if the product—“path ab”—is significantly different from zero.

As mentioned above, the direct effect (path c′) reflects the adjusted relation between intention and agency after the effect of the indirect path has been accounted for. According to the logic of mediation analysis (MacKinnon, 2008), if the magnitude of the direct effect (path c′) is significantly reduced relative to the magnitude of the total effect (path c), then it can be concluded that the relation between intention and agency depends on RT performance, at least to some extent (depending on the magnitude of the reduction). Such a reduction in the magnitude of direct effect would be a reasonable expectation if RT measures actually contribute relevant variance to the relation between intention and agency.

In summary, the present study has the potential to shed important new light on our understanding of how to elicit and detect VAC in the lab. First, the present study has the potential to enhance the elicitation of VAC in the lab by introducing new measures of intention and agency, as obtained within the context of the modified spatial cueing paradigm. And second, the present study has the potential to enhance the detection of VAC in the lab by using statistical mediation analyses to directly test the extent to which traditional measures of VAC based on RT performance (including the spatial cueing effect) actually accounts for this form of attention control.

## Method

Following Simmons et al. (2012), we report how we determined our sample size, all data exclusions (if any), all manipulations, and all measures in the study.

### Participants

A total of 720 participants were recruited through Prolific (www.prolific.co) in exchange for monetary payment (US $6.00). This sample size was obtained by aggregating across seven similar experiments with individual sample sizes ranging from *N* = 80 to *N* = 120. One study has been published previously (Gibson et al., 2023a), but the other six studies have not been published. We had no a priori estimates of effect size, but this sample size was sufficient to detect bivariate and partial correlations in the small range (greater than or equal to 0.10) with 0.80 power. To be included in the experiment, participants were required to (1) self-report that they were a fluent English speaker, (2) self-report normal or corrected-to-normal visual acuity, and (3) finish the experiment with an overall percentage error rate on the visual search task that was less than or equal to 30%. The Institutional Review Board at the University of Notre Dame approved all procedures reported in this manuscript.

### Stimuli and apparatus

All of the experiments reported in this study were programmed using PsychoPy Experiment Builder (Peirce et al., 2019), and virtual data collection was hosted through PsychoPy’s open science website Pavlovia. The sizing of stimuli in PsychoPy are specified in “height units,” which are *relative* to the height of participants’ computer screen while the ratio of the height to width of the stimuli remain *absolute*. The use of these units in PsychoPy ensures that stimuli are presented consistently without restricting participation based on screen-size or OS requirements. In the following description, we report the size of the stimuli in terms of height units, but for the sake of clarity, we also report their size in terms of centimeters (cm) based on a 13-in. widescreen display.

As shown in the bottom panel of Fig. 1, each trial consisted of four displays that were presented against the black background of the screen: a selection display, a fixation display, a cue display, and a target display. At the beginning of each trial, one of the four number cues was randomly assigned in the selection display, and participants were allowed to change the direction of the cue on each trial to any of the other three direction cues by pressing the *right* arrow key on the keyboard, or they could simply choose the cue that was randomly assigned. This arrow key advanced through a random sequence of the number cues, and the cycle could be repeated until a cue was selected. Participants locked in their choice by pressing the space bar, which then triggered the appearance of the fixation display followed by the cue display (which contained the selected cue) and then the target display. The fixation display contained a small white fixation dot in the center of the display; the fixation dot measured 0.015 units (0.3 cm) in diameter. Four boxes were presented 0.16 units (2.80 cm) *above*, *below*, *left of*, or *right of* central fixation. Each box appeared as a square, 0.07 units (1.30 cm) tall and 0.07 units (1.30 cm) wide, and had a black fill and gray outline. The fixation dot was replaced by the number cue that was selected in the selection display. The “1” cue referred to the *above* location, the “2” cue referred to the *right* location, the “3” cue referred to the *below* location, and the “4” cue referred to the *left* location. The number cues were 0.04 units (0.50 cm) at their widest point and 0.06 units (1.10 cm) tall. The target display contained a single white target letter (*E* or *U*) along with three nontarget letters (*A*, *P*, and *S*). Each letter was 0.04 units (0.50 cm) tall and 0.04 units (0.50 cm) wide and appeared in one of the four gray boxes.

### Procedure

Each of the three cue validity contexts (100%, 70%, and 25%) was presented in a separate block of 40 trials, and this group of three blocks was repeated four times for a total of 12 blocks (480 total experimental trials). The order of the three cue validity contexts was randomized separately within each repetition group for each participant. At the beginning of each block, participants were informed of the validity of the cues. On each trial within a block, a selection display appeared first; the selection display remained on the screen until participants made their direction choice, at which point they were instructed to press the space bar. The fixation display then appeared for 500 ms followed by the cue display. After 600 ms, the target display was added to the cue display, and the two displays remained on screen until a response was made. On each trial, the target letter was equally likely to be an *E* or *U*. Observers always pressed the “E” key with their *left* hand to discriminate the identity of the *E* target and the “U” key with their *right* hand to discriminate the identity of the *U* target.

At the end of each block, participants were instructed to rate the level of agency they felt. Participants were told that individuals are thought to have a positive sense of agency when they consider themselves to be the initiator of their actions, along with the following instructions, provided at the beginning of the experiment:

We are interested in how much agency you feel in these different visual search contexts. Please always use the cue to try to find the target, regardless of how useful or accurate it is. At the end of each block, a rating scale will appear on the screen, and you will be asked to rate the extent to which you considered yourself to be in control of finding the target. A rating of “1” will correspond to “no control,” whereas a rating of “7” will correspond to “full control.” You will respond by using the corresponding number keys on your keyboard to report the magnitude of your positive sense of agency in each block.

Of the seven experiments that were aggregated in this study, three were exact replications of the experiment as described above. The other four studies involved slight procedural changes in the selection display and/or instructions. In one of these experiments, the number cue options were restricted in the selection display such that the cue that was selected on trial *n − *1 was not an option on trial *n*. In another one of these experiments, the selection display appeared initially without a cue, and participants had to press the right arrow key at least once to get a cue. Another one of these experiments involved a slight change to the instructions such that the second sentence in the passage above regarding use of the cue was deleted. And the last experiment included both of the latter two changes. None of these slight procedural changes affected the intention, agency, and performance measures in a systemic fashion; a full accounting of these findings will be reported in a separate article to streamline the present discussion.

### Intention, agency, and performance measures

Each of these variables was repeatedly measured across the four blocks within each of the three levels of cue validity context. Note that intention and performance were both measured on each of the 480 trials, whereas agency was only measured at the end of each of the 12 blocks of 40 trials. We therefore calculated 12 intention scores and 12 performance scores for each individual (by averaging across the 40 trials within each block) to correspond with the 12 agency scores. Cronbach’s alpha was computed for each of these variables based on the consistency between these 12 scores.

Intention was operationalized as the average proportion of trials in which the direction of the cue was changed in the selection display within each block. Note that changing the cued direction beyond the initial, randomly assigned direction required at least one key press in five of the experiments (as shown in the bottom panel of Fig. 1), but it required at least two key presses in two of the experiments because the selection display appeared blank initially and the first key press only revealed the randomly assigned cue. In other words, with respect to Fig. 1, the first key press would have delivered the “3” cue (and it was considered equivalent to no key presses in the original version of the experiment), and the second key press would have delivered the “1” cue (and it was considered equivalent to one key press in the original version of the experiment). Regardless of this slight procedural difference, this variable was interpreted as a measure of the strength of participants’ desire to find the target in a particular location as well as the commitment to shift their attention in that direction, with higher proportions representing stronger intentions. Cronbach’s alpha was found to be 0.970 for the intention variable.

Agency was directly measured by registering participants’ self-reported agency ratings at the end of each block using a 7-point scale. This variable was interpreted as a measure of the extent to which participants felt in control of their actions, with higher scores reflecting a greater sense of agency. Cronbach’s alpha was found to be 0.887 for the agency variable.

Because the spatial cueing effect cannot be measured in the 100%-valid cue context, we considered two separate measures of performance in the present study. For the first measure of performance, we operationalized performance as the average time it took participants to correctly discriminate the identity of the target letter within each block, which included both valid and invalid trials when cue validity was less than 100%. This measure was calculated in all three cue validity contexts. Cronbach’s alpha was found to be 0.982 for the overall RT variable.

For the second measure of performance, we operationalized performance as the spatial cueing effect (invalid RTs − valid RTs), and it only included the 70%-valid and 25%-valid cue contexts. Thus, the reliability of this variable was based on only 8 scores. Cronbach’s alpha was found to be 0.750 for the spatial cueing effect variable.

Both RT measures were interpreted to reflect the strength of participants’ intentions to use the cues to shift their attention, with faster overall RTs and larger spatial cueing effects reflecting stronger intentions. Both RT measures of performance were measured in seconds so that these scores were similar in range to the intention scores. Note that, for both measures, only correct RTs were included; in addition, mean correct RTs less than 0.15 s or greater than 4.0 s were excluded from analysis (1.71%) as were mean correct RTs ± 3.0 standard deviations from the overall mean of each participant (1.94%).

## Results

**Fig. 3:**
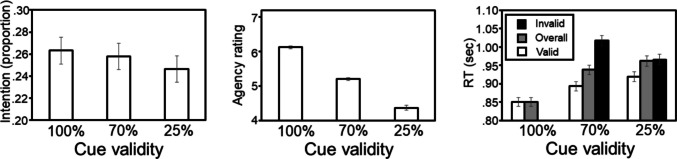
Mean intention, agency, and performance scores depicted as a function of cue validity context. Error bars represent the standard error

Figure 3 shows the average intention, agency, and RT scores depicted as a function of cue validity context. For all three measures, we expected scores to be moderated by cue validity context. As can be seen in Fig. 3, the main effect of cue validity context was significant for all three measures: for intention scores, *F*(2, 718) = 6.57, *p* = 0.001, η^2^ = 0.02; for agency scores, *F*(2, 718) = 301.00, *p* < 0.001, η^2^ = 0.46; for overall RT scores, *F*(2, 718) = 188.44, *p* < 0.001, η^2^ = 0.34; and for the spatial cueing effect (invalid RT − valid RT), *F*(1, 719) = 206.29, *p* < 0.001, η^2^ = 0.22. (Note that the main effect of cue validity context was not significant for either performance measure when error rates were analyzed, both *p* values > 0.11 and greater.)

Although the analyses reported above focused on group averages, the primary goal of the present study was to use statistical mediation models to examine the extent to which individual differences in intention scores covaried with agency and RT scores within and across each of the three cue validity contexts. The bivariate correlations between intention, agency, and performance are listed in Table 1 in each of the three cue validity contexts separately. All the mediation analyses reported below were conducted using SAS PROC MIXED. Model parameters were estimated using restricted maximum likelihood (REML), and accompanying *p* values were calculated based on a Wald test, both of which are the default setting in SAS PROC MIXED. Note that all path weights reported below represent unstandardized estimates in the original units of the dependent variable.

**Table 1:** Bivariate correlations between intention, agency, overall RT, and the spatial cueing effect (SCE) listed as a function of cue validity context

	1. Intention	2. Agency	3. Overall RT	4. SCE
	100% valid			
1. Intention	—			
2. Agency	.124***	—		
3. Overall RT	.102**	− .142***	—	
4. SCE	NA	NA	NA	—
	70% valid			
1. Intention	—			
2. Agency	− .030	—		
3. Overall RT	.176***	− .107**	—	
4. SCE	.241***	− .181***	.233***	—
	25% valid			
1. Intention	—			
2. Agency	− .113**	—		
3. Overall RT	.264***	− .131***	—	
4. SCE	.015	− .055	.049	—

^*^p ≤ .05; **p ≤ .01; ***p ≤ .001

**Fig. 4:**
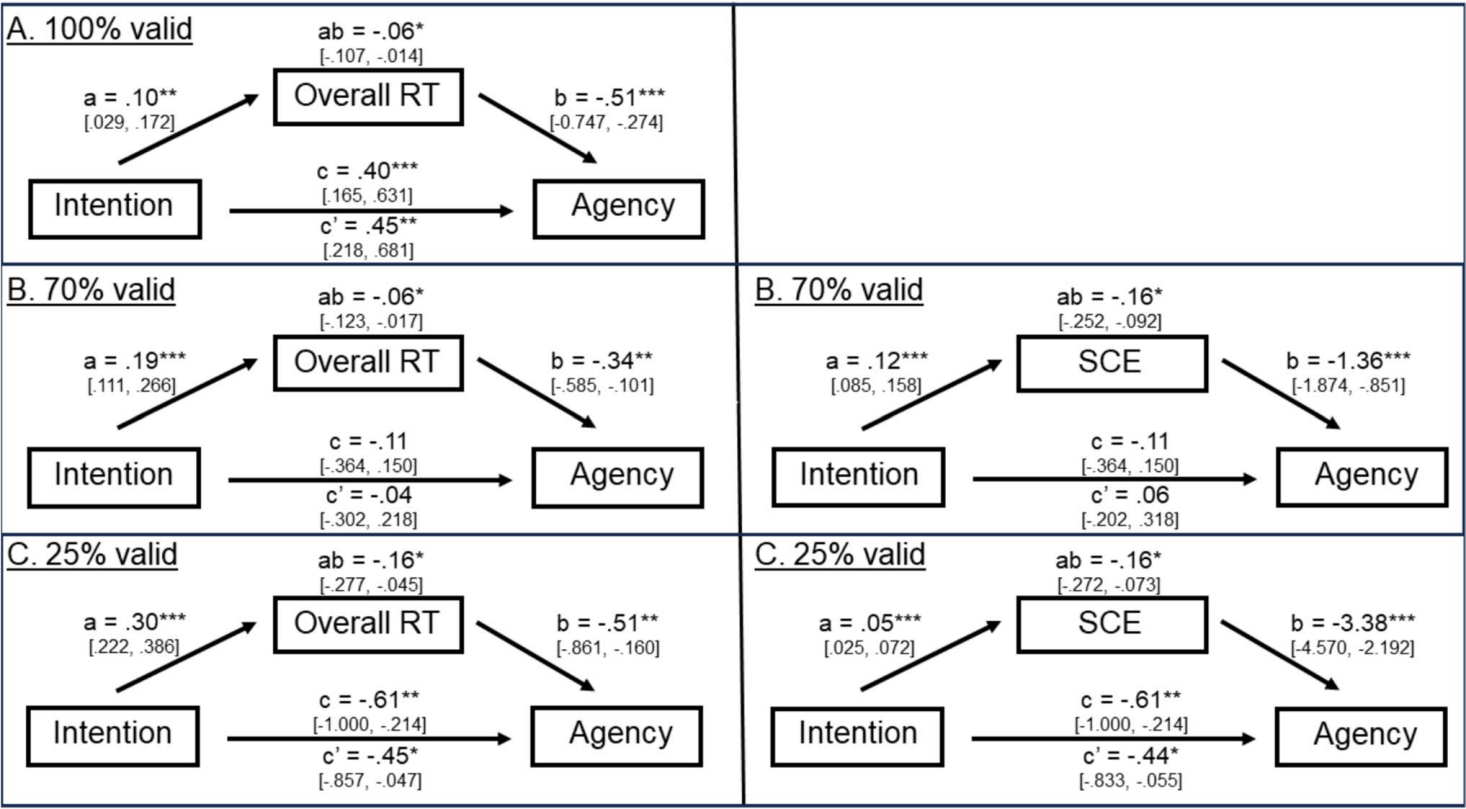
Results of the mediation analysis for each of the three cue validity contexts. The panel on the left included overall RT as the measure of performance, and the panel on the right included the spatial cueing effect as the measure of performance. All path weights represent unstandardized estimates in the original units of the dependent variable. *p ≤ .05; **p ≤ .01; ***p ≤ .001

The results of the statistical mediation analysis are shown in Fig. 4 in each of the three cue validity contexts. The left-hand panel of Fig. 4 shows these models when performance was operationalized as overall RT and the right-hand panel of Fig. 4 shows these models when performance was operationalized as the spatial cueing effect.

### The total effect of intention on agency (path c)

This analysis examined the relation between intention and agency. We predicted that there should generally be a positive relation between intention and agency at the individual level (stronger intentions should be associated with stronger agency). But we also predicted that the relation between intention and agency should be moderated by the experimental manipulation of cue validity context, because this manipulation reflects the amount of consistency between participants’ intentions and the external task context. Thus, it is reasonable to expect that the association between intention and agency should be more positive in the 100%-valid cue context than in the 25%-valid cue context. The statistical model for path c included intention, cue validity context, and intention × cue validity context as the independent variables, and it included agency as the dependent variable. Note that cue validity context was treated as a categorical variable in all the analyses reported in this article, whereas the other independent variables were treated as continuous variables. In addition, note that all the models reported in this article included a random intercept factor.

Consistent with expectation, although the main effect of intention was not significant, *F*(1, 863) = 0.39, *p* = 0.53, the intention × cue validity context interaction was significant, *F*(2, 1454) = 233.88, *p* < 0.0001, indicating that the positive association between intention and agency observed in the 100%-valid cue context became weaker and more negative as cue validity context decreased. As shown in both columns of Fig. 4, the values of “path c” in each of the three cue validity contexts were as follows: agency increased significantly (by 0.40 units) for every proportional increase in intention in the 100%-valid cue context, *F*(1, 718) = 11.24, *p* = 0.0008, 95% CI [0.165, 0.631]; in contrast, agency decreased non-significantly (by − 0.11 units) in the 70%-valid cue context, *F*(1, 718) = 0.67, *p* = 0.42, 95% CI [− 0.364, 0.150], and it decreased significantly (by − 0.61 units) in the 25%-valid cue context, *F*(1, 718) = 9.21, *p* = 0.002, 95% CI [− 1.000, − 0.214], for every proportional increase in intention.

### The indirect effect of intention on agency (path a)

This analysis examined the relation between intention and each of the two measures of RT performance. With respect to the relation between intention and overall RT, we predicted that there should generally be a negative relation between intention and RT at the individual level (stronger intentions should be associated with smaller or faster overall RTs), and this relation should also be moderated by cue validity context. That is, it is reasonable to expect that the association between intention and RT should be more negative in the 100%-valid cue context than in the 25%-valid cue context. The statistical model for this version of “path a” included intention, cue validity context, and intention × cue validity context as the independent variables, and it included overall RT as the dependent variable.

There was a significant main effect of intention, *F*(1, 2088) = 31.70, *p* < 0.0001, but contrary to expectation, the results indicated that intention was positively associated with overall RT. In other words, overall RTs actually got slower as intentions got stronger. Moreover, this positive association became stronger as cue validity context deceased, *F*(2, 1439) = 243.53, *p* < 0.0001, though it was significant in each of the three cue validity contexts. As shown in the left-hand panel of Fig. 4, the values of “path a” in each of the three cue validity contexts were as follows: the overall RT increased significantly (by 0.10 s) in the 100%-valid cue context, *F*(1, 718) = 7.61, *p* = 0.006, 95% CI [0.029, 0.172], it increased significantly (by 0.19 s) in the 70%-valid cue context, *F*(1, 718) = 22.80, *p* < 0.0001, 95% CI [0.111, 0.266], and it increased significantly (by 0.30 s) in the 25%-valid cue context, *F*(1, 718) = 53.45, *p* < 0.0001, 95% CI [0.222, 0.386], for every proportional increase in intention.

With respect to the relation between intention and the spatial cueing effect, we predicted that there should generally be a positive relation between intention and the spatial cueing effect at the individual level (stronger intentions should be associated with larger spatial cueing effects), and this relation should also be moderated by cue validity context. That is, it is reasonable to expect that the association between intention and the spatial cueing effect should be more positive in the 70%-valid cue context than in the 25%-valid cue context. The statistical model for this version of “path a” included intention, cue validity context, and intention × cue validity context as the independent variables, and it included the spatial cueing effect as the dependent variable.

Consistent with expectation, there was a significant main effect of intention, *F*(1, 808) = 44.26, *p* < 0.0001, indicating that intention was positively associated with the spatial cueing effect. In other words, the magnitude of the spatial cueing effect got larger as intentions got stronger. Moreover, this positive association became weaker as cue validity context deceased, *F*(1, 731) = 147.35, *p* < 0.0001, though it was significant in each of the two cue validity contexts. As can be seen in the right-hand panel of Fig. 4, the values of “path a” in each of the two cue validity contexts were as follows: the magnitude of the spatial cueing effect increased significantly (by 0.12 s) in the 70%-valid cue context, *F*(1, 718) = 43.36, *p* < 0.001, 95% CI [0.085, 0.158], and it increased significantly (by 0.05 s) in the 25%-valid cue context, *F*(1, 718) = 15.99, *p* < 0.0001, 95% CI [0.025, 0.072], for every proportional increase in intention.

### The indirect effect of intention on agency (path b)

This analysis examined the relation between each of the two measures of performance and agency. With respect to the relation between overall RT and agency, we predicted that there should generally be a negative relation between overall RT and agency at the individual level (faster overall RTs should be associated with stronger agency), and this relation should also be moderated by cue validity context. That is, it is reasonable to expect that the association between overall RT and agency should be more negative in the 100%-valid cue context than in the 25%-valid cue context. Note, as is customary in statistical mediation analysis (MacKinnon, 2008), we examined “path b” and “path c′” simultaneously within the same model. This allows examination of the effect of overall RT on agency while controlling for the effect of intention on agency (path b), and it allows examination of the effect of intention on agency while controlling for the effect of overall RT on agency (path c′). The statistical model for this version of “path b” and “path c′ “included intention, overall RT, cue validity context, intention × cue validity context, and overall RT × cue validity context as the independent variables, and it included agency as the dependent variable. This section will focus on those aspects of the model that reflect “path b.”

Consistent with expectation, there was a significant main effect of overall RT when the effects of intention were controlled,* F*(1, 930) = 38.37, *p* < 0.0001, indicating that overall RT was negatively associated with agency. In other words, agency ratings got stronger as overall RT got faster. In addition, there was a significant overall RT × cue validity context interaction when the effects of intention were controlled,* F*(2, 1451) = 163.18, *p* < 0.0001, indicating that this relation varied across cue validity context. As can be seen in the left-hand panel of Fig. 4, the values of “path b” in each of the three cue validity contexts were as follows: agency decreased significantly (by − 0.51 units) in the 100%-valid cue context, *F*(1, 717) = 17.98, *p* < 0.0001, 95% CI [− 0.747, − 0.274], it decreased significantly (by − 0.34 units) in the 70%-valid cue context, *F*(1, 717) = 7.77, *p* = 0.006, 95% CI [− 0.585, − 0.101], and it decreased significantly (by − 0.51 units) in the 25%-valid cue context, *F*(1, 717) = 8.17, *p* = 0.004, 95% CI [− 0.861, − 0.160], for every one second increase in overall RT.

With respect to the relation between the spatial cueing effect and agency, we predicted that there should generally be a positive relation between the spatial cueing effect and agency at the individual level (larger spatial cueing effects should be associated with stronger agency), and this relation should also be moderated by cue validity context. That is, it is reasonable to expect that the association between the spatial cueing effect and agency should be more positive in the 70%-valid cue context than in the 25%-valid cue context. The statistical model for this version of “path b” and “path c′” included intention, the spatial cueing effect, cue validity context, intention × cue validity context, and the spatial cueing effect × cue validity context as the independent variables, and it included agency as the dependent variable. This section will focus on those aspects of the model that reflect “path b.”

There was a significant interaction between the spatial cueing effect and cue validity context when the effects of intention were controlled, *F*(1, 795) = 139.88, *p* < 0.0001, but contrary to expectation, the results indicated that the spatial cueing effect was negatively associated with agency in both contexts. In other words, agency actually got weaker as the magnitude of the spatial cueing effect got stronger, and it was significantly negative in both contexts. As can be seen in the right-hand panel of Fig. 4, the values for “path b” in each of the two cue validity contexts were as follows: agency decreased significantly (by − 1.36 units) in the 70%-valid cue context, *F*(1, 717) = 27.35, *p* < 0.001, 95% CI [− 1.874, − 0.851], and it decreased significantly (by − 3.38 units) in the 25%-valid cue context, *F*(1, 717) = 31.14, *p* < 0.0001, 95% CI [− 4.570, − 2.192], for every 1-s increase in the magnitude of the spatial cueing effect.

### Evaluating the significance of the overall indirect effect (path ab)

Altogether, analyses of the overall indirect path revealed that “path ab” was significantly *negative* in all three cue validity contexts when overall RT served as the performance measure. As can be seen in the left-hand panel of Fig. 4, the values of “path ab” in each of the three cue validity contexts were as follows: path ab = − 0.06, *SE* = 0.024, 95% CI [− 0.107, − 0.014] in the 100%-valid cue validity context, path ab = − 0.06, *SE* = 0.027, 95% CI [− 0.123, − 0.017] in the 70%-valid cue validity context, and path ab = − 0.16, *SE* = 0.059, 95% CI [− 0.277, − 0.045] in the 25%-valid cue validity context.

Likewise, analyses of the overall indirect path revealed that “path ab” was significantly *negative* in both cue validity contexts when the spatial cueing effect served as the performance measure. As can be seen in the right-hand panel of Fig. 4, the values of “path ab” in each of the two cue validity contexts were follows: path ab = − 0.16, *SE* = 0.041, 95% CI [− 0.252, − 0.092] in the 70%-valid cue validity context; and path ab = − 0.16, *SE* = 0.051, 95% CI [− 0.272, − 0.073] in the 25%-valid cue validity context.

### The direct effect of intention on agency (path c′)

This analysis examined the relation between intention and agency when the relation between RT performance and agency was controlled, and it used the same statistical models that were used to examine “path b.” Above we showed that the indirect effect of intention on agency (path ab) was significant regardless of whether overall RT or the spatial cueing effect served as the measure of performance. If the indirect effect accounts for any of the variance in the total effect, then the magnitude of the direct effect (path c′) should be reduced when the relation between RT performance and agency was controlled.

Similar to the total effect, the intention × cue validity context interaction was significant when the effects of overall RT were controlled, *F*(2, 1446) = 18.89, *p* < 0.0001. As can be seen in the left-hand panel of Fig. 4, the values of “path c′” in each of the three cue validity contexts were as follows: agency increased significantly (by 0.45 units) in the 100%-valid cue context, *F*(1, 717) = 14.51, *p* = 0.0002, 95% CI [0.218, 0.681], for every proportional increase in intention; in contrast, agency decreased nonsignificantly (by − 0.04 units) in the 70%-valid cue context, *F*(1, 717) = 0.10, *p* = 0.75, 95% CI [− 0.302, 0.218], and it decreased significantly (by − 0.45 units) in the 25%-valid cue context, *F*(1, 717) = 4.80, *p* = 0.03, 95% CI [− 0.857, − 0.047], for every proportional increase in intention. Notice that in each of these three contexts, the magnitude of “path c′” was actually larger (more positive) than the magnitude of “path c.” As MacKinnon et al. (2000) have shown, the significance of the difference between “path c” and “path c′” is equivalent to the significance of “path ab” reported above (i.e., path ab = path c − path c′). Thus, we can conclude that the magnitude of “path c′” was significantly larger (more positive) than the magnitude of “path c” in each of the three cue validity contexts.

Likewise, similar to the total effect, the intention × cue validity context interaction was significant when the effects of spatial cueing were controlled, *F*(1, 701) = 43.33, *p* < 0.0001. As can be seen in the right-hand panel of Fig. 4, the values of “path c′” in each of the two cue validity contexts were as follows: agency increased non-significantly (by 0.06 units) in the 70%-valid cue context, *F*(1, 717) = 0.19, *p* = 0.66, 95% CI [− 0.202, 0.318], and it decreased significantly (by − 0.44 units) in the 25%-valid cue context, *F*(1, 717) = 31.14, *p* < 0.0001, 95% CI [− 0.833, − 0.055], for every proportional increase in intention. Similar to overall RT, the magnitude of “path c′” was significantly larger (more positive) than the magnitude of “path c.”

## General discussion

The present study attempted to increase the elicitation of VAC by using a modified spatial cueing paradigm that granted participants greater freedom in choosing the direction of the cue (Gibson et al., 2023a). It attempted to improve the detection of VAC by examining the relations between intention, agency, and RT performance as well as how the magnitude of these relations might be moderated by cue validity context. A central goal of the present study was to utilize statistical mediation analysis to gain a deeper understanding of VAC by examining the total, indirect, and direct effects of intention on agency.

Examination of the total effect revealed that a positive association between intention and agency could be observed in this paradigm, but the results also suggested that this association was moderated by cue validity context. As expected, the total effect was found to be significantly *positive* in the 100%-valid cue context, but it was found to be non-significant in the 70%-valid cue context, and it was found to be significantly *negative* in the 25%-valid cue context. We interpret the significant positive total effect of intention on agency that was observed in the 100%-valid cue context to be a manifestation of VAC in this paradigm, and we sought to explain this total effect by examining the nature of the indirect effect.

Specifically, examination of the indirect effect revealed that the effect of intention on agency was mediated by RT performance. However, this indirect effect was contrary to expectation. Consider first the results when RT performance was operationalized as overall RT. With respect to “path a,” stronger intentions were found to be positively associated with overall RTs across all three cue validity contexts (though the magnitude of this path did increase as cue validity decreased). These findings are important because they suggest that increases in the strength of intention can actually slow performance on speeded RT tasks regardless of whether the task context is favorable or unfavorable to fulfilling the intention, and thus regardless of whether there are any invalid trials or not. Although this pattern of findings was unexpected in the present context, they do corroborate other findings which have concluded that “the speed of free will” is slow (Horowitz et al., 2009; Wolfe et al., 2000), though it should be noted that these previous studies did not really attempt to increase agential capacity as we have defined it (List, 2019). Moreover, these previous studies tended to focus solely on “path a,” and they did not consider “path b,” which examines the extent to which RT performance might be related agency—the feeling of being in control. With respect to “path b,” slower RTs were in turn found to be negatively associated with weaker feelings of agency, as expected. Altogether, analyses of the overall indirect path revealed that “path ab” was significantly *negative* in all three cue validity contexts.

Similar findings were obtained when RT performance was operationalized as the spatial cueing effect. With respect to “path a,” as expected, stronger intentions were found to be positively associated with spatial cueing effects across both the 70%-valid and 25%-valid cue contexts (though the magnitude of this path did decrease as cue validity decreased). These findings are important because they provide evidence at the individual level that increases in the strength of intention can generate larger spatial cueing effects within a cue validity context, which lends support to the validity of our measure of intention at the individual level of analysis. However, larger spatial cueing effects were unexpectedly found to be negatively associated with agency when “path b” was examined. Moreover, similar to our analysis of overall RT, analyses of the overall indirect path revealed that “path ab” was significantly *negative* in both cue validity contexts.

The fact that the direction of the indirect path—path ab—was found to be significantly *negative* for both measures of performance and across all three cue validity contexts therefore leads to the counterintuitive conclusion that increases in the strength of intention are associated with decreases in the sense of agency when the effects of intention are channeled through speeded performance measures. As such, these findings are reminiscent of “ironic processes of mental control” (Wegner, 1994). As Wegner pointed out, attempts to use intention to engage in volitional behaviors can sometimes lead to the opposite, counter-volitional behavior. For instance, an intention to not think of the white bear can sometimes lead to an increase in such thoughts, reflecting a failure of mental control (Wegner, 1989; Wegner et al., 1987). Although the present findings are perhaps more nuanced than Wegner’s original findings, they are nevertheless notable in showing that the same intentions that can lead to a significant *increase* in the total effect of intention on agency in the 100%-valid cue context, can simultaneously lead to a significant *decrease* in agency in that same context when those intentions are channeled through RT performance. This finding is important because speeded performance measures such as the spatial cuing effect have served as the standard behavioral markers of VAC since the inception of the spatial cueing paradigm (see, e.g., Posner et al., 1980). However, the present findings suggest that the use of such measures may not be warranted because they actually reflect the opposite of VAC when a more complete picture of volitional action is provided (see Trost & Gibson, 2024, for another critique of the spatial cueing effect). We will return to this issue below, but first we consider the direct effect of intention on agency.

Examination of the direct effect revealed that the association between intention and agency was also moderated by cue validity context. Similar to the total effect, the direct effect was found to be significantly *positive* in the 100%-valid cue context. In contrast, the direct effect was found to be nonsignificant and close to zero in the 70%-valid cue context, and it was found to be significantly *negative* in the 25%-valid cue context.

Note, however, that contrary to expectation, the magnitude of the direct effect was not reduced in the 100%-valid cue context when RT performance was controlled. In fact, it actually grew more positive, which made it become larger relative to the total effect (it increased from 0.40 to 0.45 units of agency per unit increase in intention). This type of mediation is typically referred to as “inconsistent mediation,” suggesting that the performance variable actually functioned more as a suppressor variable in this cue validity context than as a mediator (MacKinnon et al., 2000). This finding is consistent with our interpretation that the indirect path reflected a form of counter-will that operated in opposition to the direct path, which reflected the will.

On the one hand, the fact that the magnitude (and direction) of the direct effect of intention on agency was dependent on cue validity context is consistent with conventional wisdom, as it has long been thought that VAC should be mostly confined to informative cue validity contexts such as the 100%-valid and 70%-valid cue contexts (see Theeuwes, 2018, for a review). As such, we interpret the positive direct effect of intention on agency that was observed in the 100%-valid cue context as a manifestation of VAC. On the other hand, although this conclusion is consistent with conventional wisdom, it is important to realize that this finding was obtained in the modified spatial cueing paradigm and would not have been expected to occur in the standard spatial cueing paradigm, where there are no independent measures of intention and the evidence for the expression of agential capacity has been found to be weak and inconsistent (Gibson et al., 2023a, 2023b). Moreover, this direct effect was observed after the contrary effects of the indirect path involving standard measures of performance were controlled. Thus, it is important to acknowledge that the present findings do not simply support the status quo in the attention control literature. It should also be pointed out that evaluation of the direct effect of intention on agency provided no evidence for VAC in the 70%-valid cue context, despite the presence of informative cues.

Before closing, we would like to mention one potential liability of the modified spatial cueing paradigm. Although the main point of the present study was to increase participants’ agential capacity by allowing them to freely change the cued direction, many participants exercised their capacity by never choosing to change the cued direction. Table 2 documents these extreme cases by first separating trials into two “choice” contexts within each of the three cue validity contexts—namely, one choice context in which cued direction was changed, and another choice context in which cued direction was unchanged.^1^ As can be seen in the choice column of the lower half of Table 2, the percentage of participants who never changed the cued direction (and thus had missing data in the “cued direction changed” context) ranged from 21.80% in the 100%-valid cue context to 38.33% in the 25%-valid cue context. In contrast, as can be seen in the choice column of the upper half of Table 2, the percentage of participants who always changed the cued direction (and thus had missing data in the “cued direction unchanged” context) ranged from 0.42% in the 100%-valid cue context to 1.80% in the 25%-valid cue context. In addition, Table 2 also lists the number of cases with missing data in each choice context that was due to RT trimming and error rates; these missing cases occurred because the participant chose the context infrequently and then either responded too slowly or committed 100% errors on those trials in which the infrequent context was chosen.

**Table 2:** Number of participants (out of 720) with missing values due to lack of context choice, RT trimming, or error rates in the valid and invalid conditions for each of the three cue validity contexts listed as a function of choice context

	Cued direction unchanged							
	Valid condition				Invalid condition			
	Choice	Trimmed	Errors	Total	Choice	Trimmed	Errors	Total
100%	3	1	0	4	NA	NA	NA	NA
70%	6	1	0	7	10	0	0	10
25%	13	0	0	13	4	1	0	5
	Cued direction changed							
	Choice	Trimmed	Errors	Total	Choice	Trimmed	Errors	Total
100%	157	18	9	184	NA	NA	NA	NA
70%	193	19	3	215	248	32	5	285
25%	276	16	4	296	200	30	5	235

Missing values due to RT trimming or errors occurred because participants chose the context infrequently and then either responded too slowly or committed 100% errors on those trials in which the infrequent context was chosen

**Fig. 5:**
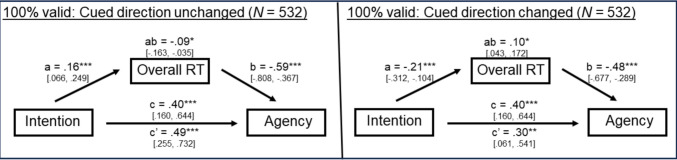
Results of the mediation analysis in the 100%-valid cue context for each of the two choice contexts. The panel on the left depicts the cued direction unchanged context, and the panel on the right depicts the cued direction changed context. All path weights represent unstandardized estimates in the original units of the dependent variable. *p ≤ .05; **p ≤ .01; ***p ≤ .001

Given that some participants only experienced one choice context or the other, we decided to reexamine the total, indirect, and direct effects in our mediation model when these extreme participants were excluded from the analysis. In addition, we also reexamined these three effects within each choice context separately (i.e., “cued direction changed” and “cued direction unchanged” contexts). Here we will focus exclusively on the 100%-valid cue context because this context provided the strongest evidence for VAC. The bivariate correlations between intention, agency, and overall RTs in the “cued direction changed” and “cued direction unchanged” contexts are listed in Table 3, and the corresponding mediation models for each choice context are shown in Fig. 5. Note that we also considered testing both overall RT measures within the same, two-mediator model, but ultimately decided against this model due to the high correlation between these two measures (*r* = 0.756; see Table 3), which would have likely obfuscated the relation between each type of overall RT and agency (path b).

**Table 3:** Bivariate correlations in the 100%-valid cue context between intention, agency, overall RT in the cued direction changed (C) condition, and overall RT in the cued direction unchanged (U) condition

	Intention	Agency	Overall RT C	Overall RT U
1. Intention	—			
2. Agency	.140***	—		
3. Overall RT C	− .168***	− .227***	—	
5. Overall RT U	.146***	− .196***	.756***	—

Note that these correlations are based on a subset of the original sample (*N* = 532) that excludes participants who either always changed cued direction or never changed cued direction

These correlations are based on a subset of the original sample (*N* = 532) that excludes participants who either always changed cued direction or never changed cued direction. *p ≤ .05; **p ≤ .01; ***p ≤ .001

The total, indirect, and directs effects obtained in the “cued direction unchanged” context (see the left-hand panel of Fig. 5) replicated the main results that were summarized in Fig. 4 and discussed above. However, although the results obtained in the “cued direction changed” context (see the right-hand panel of Fig. 5) were also very similar to the main results, there was a critical change in the direction of the relation between intention and overall RT (path a). In particular, this path was now significantly *negative* indicating that stronger intentions were associated with faster overall RTs. As a result, the overall “ab path” was also positive, which in turn made the sign of the indirect path consistent with the sign of the direct path, and the interpretation of both paths was now aligned in that both suggested that stronger intentions were associated with stronger agency. As expected, the direct effect of intention on agency was now significantly reduced (from 0.40 to 0.30 units of agency per unit change in intention), when the indirect effect of intention on agency was controlled, but the direct effect remained significant.^2^

Although the visual search task was identical regardless of whether participants chose to change cued direction or not at the start of each trial, it is interesting that speeded task performance was sensitive to this choice context (cf. Gopher et al., 2000). Given that intention scores reflected the proportion of trials in which participants changed cued direction, one way to explain the sign change in “path a” across the two choice contexts is that the relation was negative when high intention scores reflected relatively more experience in the chosen context (i.e., the “cued direction changed” context), whereas this relation was positive when high intention scores reflected relatively less experience in the chosen context (i.e., the “cued direction unchanged” context).^3^ Thus, the aspect of intention that is related to agency through the indirect path might be based more on experience (see, e.g., Anderson et al., 2021; Trost & Gibson, 2024), whereas the aspect of intention that is related to agency through the direct path might be based more on volition. If this analysis is correct, these two aspects of intention would be linked to different forms of attention control—one based more on experience and the other based more on volition. Indeed, it is important to point out that only small changes were observed in the magnitude of the direct effect of intention on agency (relative to the total effect), after the indirect effect was controlled, in any of the models shown in Figs. 4 and 5. These findings are important because they suggest that the more experience based indirect path did not account for all of the variance in the more volitional based direct path.

In conclusion, the present findings advance theoretical understanding of VAC by providing preliminary evidence for a dual-path model. The “direct effect” of intention on agency generally reflects VAC in that increases in intention were associated with increases in agency, but only in the 100%-valid cue context. However, the “indirect effect” of intention on agency passes through performance, and it reflects a process that is different from, and sometimes operates counter to VAC. Preliminary exploratory analyses suggested that the indirect path may be based more on experience whereas the direct path may be based more on volition. Altogether, the present study recommends new methods for eliciting and detecting VAC in the lab while also exposing some shortcomings in more traditional measures of VAC based on performance.

## Data Availability

All data reported in the present study are available at https://osf.io/8d3qn/.

## Footnotes

1 We thank Michael Kane for this suggestion.

2 Not surprisingly, “path a” was estimated to be smaller (0.07), and it was found to be nonsignificant (*p* = 0.12) for this subset of 532 participants when overall RT was averaged across choice context (as it was in the main analyses). This can now be understood to reflect that the sign of “path a” was opposite across the two choice contexts.

3 Note that it might be tempting to think that the sign of “path a” could also be made negative in the “cued direction unchanged” context simply by reverse scoring the intention measure in this model. However, while doing this would change the sign of “path a” from positive to negative (which in turn would make the product of “path a” and “path b” positive), it would also change the sign of “path c′” (and path c) from positive to negative. Thus, the signs of the direct (negative) and indirect (positive) paths would still be inconsistent in this model.

## Code availability

All SAS code used to analyze the data is available at https://osf.io/8d3qn/.

## Declarations

### Open practices

All data and SAS code are available at https://osf.io/8d3qn/. None of the experiments were preregistered*.*
